# Bipolar offspring and mothers: interactional challenges at infant age 3 and 12 months—a developmental pathway to enhanced risk?

**DOI:** 10.1186/s40345-020-00192-3

**Published:** 2020-08-31

**Authors:** Teija M. S. Anke, Kari Slinning, Vibeke Moe, Cathrine Brunborg, Torill S. Siqveland, Dag Vegard Skjelstad

**Affiliations:** 1 Division of Mental Health and Addiction, Vestre Viken Hospital Trust, 3004 Drammen, Norway; 2 The Center for Child and Adolescent Mental Health, Eastern and Southern Norway, Oslo, Norway; 3 Department of Psychology, University of Oslo, Oslo, Norway; 4 Oslo Centre for Biostatistics and Epidemiology, Research Support Services, Oslo University Hospital, Ullevål, Oslo, Norway; 5 Oslo University Hospital, Ullevål, Oslo, Norway

**Keywords:** Bipolar disorder, Offspring, Mother–infant interaction, Prospective study, Developmental pathway, Dyadic coordination, At-risk states

## Abstract

**Background:**

Bipolar offspring are considered a high-risk group for developing mental disorders. Developmental outcomes result from additive and interactive effects of biological vulnerability and environmental influences. Mother–infant interactions represent important early environmental influences that may modify infants’ risk of mental disorders. The aim of the current prospective study was to investigate the patterns and development of mother–infant interactions in the first year of life in dyads in which the mothers have bipolar disorder (BD).

**Methods:**

Twenty-six dyads in which the mothers had BD and 28 dyads in which the mothers had no mental disorder were video-taped in a free play interaction. The Parent–Child Early Relational Assessment (PCERA) was used to assess the quality of the interactions on three domains (maternal behaviour, infant behaviour and dyadic coordination) at 3 and 12 months of infant age. First, we compared the mother–infant interaction patterns between the two groups at 12 months. Second, we investigated how the patterns developed within and between the groups from infant ages 3 to 12 months.

**Results:**

BD dyads demonstrated significantly more challenges in all three interaction domains at infant age 12 months compared to the healthy dyads. This observation was in line with the findings at infant age 3 months. Subdued expression of positive affect and mutual underinvolvement represented core challenges in maternal and infant behaviours in the BD dyads. Continuous difficulties with dyadic coordination and reciprocity were the most concerning interaction behaviours at 3 and 12 months. On the positive side, there was little expression of negative affect or tension in maternal, infant and dyadic behaviour, and some positive changes in infant behaviour from 3 to 12 months.

**Conclusions:**

The current results suggest that challenges in mother–infant interaction patterns in the first year of life may enhance the developmental risk for bipolar offspring. Clinical interventions should address both the BD mothers’ needs in relation to postpartum mood deviations and mother–infant interactions. We suggest interaction interventions to promote dyadic coordination and reciprocity, such as helping mothers being more sensitive to their infant’s cues and to provide attuned contingent responses.

## Background

It is well established that bipolar offspring are a high-risk group for developing mental disorders. Estimates indicate that the risk of developing bipolar disorder (BD) is between 6 and 9% (Rasic et al. 2013; Smoller and Finn, 2003), with a broader risk of 60% for any mental disorder when one of the parents has BD (Rasic et al. 2013). Current research and theoretical models emphasise that developmental outcomes result from additive and interactive effects of biological vulnerability and environmental influences (Chang et al. 2003; McGowan and Kato 2008; Willcutt and McQueen 2010). Heritability is estimated to explain 60 to 85% of the variance in risk (Smoller and Finn 2003). Nevertheless, the majority of bipolar offspring do not develop BD, and many do not develop any mental disorder (Rasic et al. 2013). In line with a developmental psychopathology framework, different influences need to be considered at different stages to understand the development of mental health risk and resilience (Cicchetti 2010; Willcutt and McQueen 2010).

In early life, caregiver-infant interactions contribute as environmental influences with profound impact on the infant’s neurobehavioural and social-emotional development (Champagne and Curley 2005; Greenberg et al. 2014; Nelson and Bosquet 2000; Tronick 2007). Coinciding, the first postpartum year is a period with increased risk of illness relapse for women with BD (Di Florio et al. 2013; Wesseloo et al. 2016). It is estimated that postpartum women with BD have a one in two risk for any affective episode, and a one in five risk for a severe illness relapse (Di Florio et al. 2013; Jones et al. 2014). Thus, it is likely that offspring are exposed to mothers with mood symptoms during the first 12 months. However, few studies have investigated mother–infant interactions in the context of maternal BD.

Within the first year, impairments in maternal behaviour, such as reduced sensitivity, have been demonstrated among mothers with BD (Hipwell et al. 2000; Hipwell and Kumar 1996). Studies have also reported non-significant trends of decreased infant expressivity (Hipwell et al. 2000) and dyadic reciprocity (Logsdon et al. 2015).

In a recent publication we reported significant group differences in interaction patterns between mothers with and without BD and their infants at 3 months postpartum. The mothers with BD were generally positive and friendly in the interactions, but displayed more difficulties in sensitivity, involvement and contingent responsiveness than the comparison mothers, and more infants showed subdued positive affect and communication. However, the most concerning interaction behaviours were observed in mother–infant dyadic coordination (Anke et al. 2019a).

A few other studies have also reported on early interaction challenges. During admission to a specialised perinatal psychiatric care (i.e. Mother–Baby Unit), a maternal diagnosis of BD or psychosis was associated with poorer mother–infant interactions, than a maternal diagnosis of depression or anxiety disorder (Wright et al. 2018). Later in infancy and toddlerhood, avoidant infant behaviour (Gaensbauer et al. 1984), difficulties in cooperation and resolution of conflict (child ages 15–51 months) (Kochanska et al. 1987), and increased levels of insecure/disorganised attachment patterns (child ages 15–52 months) (DeMulder and Radke-Yarrow 1991) have been found in dyads in which the mother has BD.

Interactions are complex and comprise dynamic and interplaying processes between (1) parental behaviour, (2) infant behaviour, and (3) dyadic coordination (Beebe et al. 2010; Tronick 2007), implying that a comprehensive investigation ought to include all three domains. To our knowledge, we are the first to have done this in the context of BD (Anke et al. 2019a). Furthermore, to assess the gravity of interactional challenges, and the possible impact this may have on the developmental risk of bipolar offspring, it is important to get a better sense of how mother–infant interactions develop over time.

The main aim of the current study was to investigate the patterns and development of mother–infant interactions in the first year of life in dyads in which the mothers have BD, compared to dyads in which the mothers have no mental disorder. Within this main aim, our first aim was to assess mother–infant interaction patterns at infant age 12 months. Our second aim, and building on our previous findings at infant age 3 months (Anke et al. 2019a), was to investigate patterns of change in the interactions within and between the groups from infant ages 3 to 12 months. The timing of the assessments was selected to reflect important milestones in infants’ social maturation (Zeanah et al. 1997), and our decision to utilise validated, age-dependent assessment-scales when coding the mother–infant interactions. All assessments included the three interaction domains: maternal behaviour, infant behaviour and dyadic coordination. We anticipated more interaction difficulties in the BD group at 12 months, and the BD group to have developed more poorly than the comparison group between 3 and 12 months.

## Methods

### Design

This study is a prospective follow-up study of BD mothers and their infants compared with historical data from non-clinical mothers and their infants. The study is part of a larger Norwegian investigation of infant families in which the mother has BD (Anke et al. 2019a, b).

### Recruitment procedures and participants

#### BD sample

The BD sample consisted of 26 mother–infant dyads. Inclusion criteria were women in stable partner relationships with a BD I or II diagnosis who were either pregnant or had recently given birth (maximum 3 months postpartum). A cohabitating partner who was willing to participate was an inclusion criterion because of the aims of the larger investigation. The exclusion criteria were parental substance abuse, multiple-childbirth, premature birth < 35 weeks, or an infant with a known serious medical condition or syndrome.

Women were recruited from mental health outpatient clinics, infant mental health teams at child mental health services, community well-baby clinics, pregnancy care, maternity wards, through the website of the national BD association, and at group psychoeducation courses for patients with BD (Skjelstad et al. 2015). Recruitment took place between September 2014 and July 2016 in the south-eastern part of Norway.

The women’s clinical BD diagnosis was verified from their specialist mental health records and/or by contacting their specialist mental health professional and by utilising a semi-structured interview. For more details about recruitment procedures, see Anke et al. (2019b).

#### Non-clinical sample

Comparison group data of 28 mother–infant dyads were gathered from another Norwegian study. These dyads were recruited from local well-baby clinics in Oslo, Norway, between December 2004–January 2009 (Siqveland et al. 2014). Inclusion criteria were being pregnant and having no substance abuse or mental disorder. The women’s mental health status was investigated during pregnancy with the European Addiction Severity Index (McLellan et al. 1992), Millon’s Clinical Multiaxal Inventory-III (Millon 1997) and Hopkins Symptom Check List, SCL-25 (Derogatis et al. 1974). All women in the comparison group also had a cohabitating partner.

### Procedure

Data on mother–infant interactions were collected at infant ages 3 and 12 months. All interactions were video recorded. The assessed session for both samples at both time-points was a 5-min free-play interaction. The mothers were asked to interact with their infant as they typically did and as they pleased. At 3 months, there was an optional use of toys. At 12 months, the mothers were asked to actively use a selection of provided toys in the interaction.

The recordings of the BD sample were performed at the participants’ home (3 months: n = 25, 12 months: n = 24) or in a professional setting (3 months: n = 1, 12 months: n = 2). All recordings of the comparison group at 3 and 12 months were performed in a professional setting.

At the end of the video-recording session, the mothers in both samples were explicitly asked if they thought the interaction was representative. If not, the reasons were written down. One mother in the BD sample felt awkward because of the video recording at 3 months. At 12 months, two mothers in the BD sample evaluated their infants to be somewhat affected by a viral infection and being a little less active in their play than usual. The remaining mothers in both samples regarded the recorded interactions as representative.

### Assessments

#### Mother–infant interactions at infant ages 3 and 12 months

The mother–infant interactions in both samples were assessed with the Parent–Child Early Relational Assessment (PCERA) (Clark 1985, 2006, 2009, 2010). It is a standardised assessment method that has demonstrated good content, construct and factor validity, discriminant validity between clinical and non-clinical groups, as well as sensitivity to change (Clark 1985, 2006, 2009, 2010 Clark 1999; Lotzin et al. 2015). The PCERA is developed to examine strengths and concerns in parental (henceforth maternal) and infant behaviour separately and in their dyadic interactions. It contains 65 behavioural, affective and communicative variables. These are operationalised in a manual and rated numerically based on observed frequency, duration and intensity. The rating is a five-point Likert scale. The five points are categorised into three areas of concern/strength according to PCERA: 1–2 = area of concern, 3 = area of some concern and 4–5 = area of strength (Clark 1985, 2006, 2009, 2010).

In the current study, all interactions in the BD sample were rated by an independent, certified main coder. A second independent certified coder double-rated a random selection of 31% of the interactions for calculation of inter-rater reliability. A good inter-rater reliability was found using absolute agreement on ratings. Intra-class correlation was 0.75 for 3-month ratings and 0.85 for 12-month ratings. The coders were aware of the women’s BD diagnosis but were blinded to all other information.

The main coder of the BD sample also rated the interactions for the comparison group together with a second independent experienced coder. Twenty percent of randomly selected interactions were double-rated and inter-rater reliability was calculated using categorical agreement (1–2, 3, 4–5). Intra-class correlation varied between 0.80 and 0.97 at 3 months and between 0.73 and 0.94 at 12 months for the different subscales used in the study (Siqveland et al. 2014). The coders were blinded to all information about the participants.

#### PCERA subscales used for analyses

When conducting analyses on interaction data, PCERA variables were organised into subscales. Subscales were used since not all variables in the PCERA are applicable for all child ages. For the investigation of possible group differences at 12 months, we utilised a validated scale for free-play at this age (Clark 1985, 2006, 2009, 2010). The scale consists of three maternal subscales: “Maternal positive affective involvement and verbalisation” (S1), “Maternal negative affect and behaviour” (S2), “Maternal intrusiveness, insensitivity and inconsistency” (S3); three infant subscales: “Infant positive affect, communicative and social skills” (S4), “Infant quality of play, interest and attentional skills” (S5), “Infant dysregulation and irritability” (S6); and two dyadic subscales: “Dyadic mutuality and reciprocity” (S7), and “Dyadic disorganisation and tension” (S8). In all, these contained 21 maternal, 19 infant and 8 dyadic variables (see Table 1.1 in Additional file 1).

The validated 12-month scale is not suitable for investigating how interactions develop from 3 to 12 months because it contains variables that are unrateable at age 3 months. Additionally, the validated scale that was used in our previous study at 3 months (Anke et al. 2019a), does not contain all variables that are of interest at 12 months. Thus, for exploration of patterns of change in the interactions, PCERA variables that are rateable at both 3 and 12 months were clustered into subscales equivalent to behavioural, affective and communicative categories in the manual (Clark 1985, 2006, 2009, 2010). The same procedure has been used in another study (Siqveland et al. 2014). The clustered subscales comprise four maternal subscales: “Maternal tone of voice”, “Mother’s characteristic mood”, “Maternal affective and behavioural involvement”, “Maternal style”; three infant subscales: “Infant expressed affect and characteristic mood”, “Infant behavioural and adaptive abilities”, “Infant communication”, and two dyadic subscales: “Dyadic affective quality” and “Dyadic mutuality”. In all, these include 23 maternal, 18 infant and 8 dyadic variables (see Table 1.2 in Additional file 1).

For further details on the organisation of PCERA variables into subscales, see Additional file 1.

#### Maternal affective symptoms

Data on the presence of affective symptoms among mothers in the BD sample were collected in conjunction with the interaction recordings at 3 and 12 months. Depressive symptoms were assessed with the Inventory of Depressive Symptomatology (IDS) (Rush et al. 1996), and hypomanic/manic symptoms were assessed with the Young Mania Rating Scale (YMRS) (Young et al. 1978).

### Statistical analyses

Demographic and clinical data are presented as either proportions, means with their standard deviations (SD) and range, or medians with 25th and 75th percentiles.

Group differences, using PCERA mean scores on the subscales at 3 and 12 months, were analysed by independent samples t-tests. The Chi square test for contingency tables or Fisher exact test was used to detect associations between categorical variables and the BD vs. the non-clinical sample. Correlation analyses were performed separately for the BD and non-clinical samples using Pearson’s correlation coefficient (r). The Mann–Whitney U test was used to test the difference between the BD and non-clinical sample on the subscale “Infant dysregulation and irritability” (S6) at 12 months, since it was skewed.

To identify possible confounders, we studied variables that could influence the outcome, such as maternal age, education, employment status, parity, infant gestational age, infant gender, birth weight and infant exact age at interaction sessions. Only variables with significant relationships with both the exposure (BD vs. non-clinical) and the outcome variables (PCERA maternal, infant and dyadic subscales) at 12 months were considered possible confounders and included in the multiple linear regression analysis. Median regression was applied to adjust for confounding factors when studying subscale 6 at 12 months, since it was skewed.

Paired sample t-tests were used to estimate the mean change from 3 months to 12 months within the BD and the non-clinical sample on the clustered subscales. Independent sample t-tests were conducted to test whether the mean change on any clustered subscale measures from 3 to 12 months differed between the BD and the non-clinical sample. Multiple linear regression analyses were performed to test for differences in the mean change between groups while adjusting for confounding factors. Confounding factors were identified using the same procedure as described above.

Significant confounders in the current study were maternal age and maternal employment status. (See “Results” section for details on confounding effects.)

Pearson correlation analyses and linear regression analyses were used to examine the association between concurrent maternal symptom load and the outcome variables.

Overall, a significance level of 0.05 was used. Effect sizes were calculated by Cohen’s *d* or the correlation coefficient *r*. For Cohen’s *d*, small effect sizes were defined as 0.20, medium as 0.50 and large as 0.80 and higher (Ellis 2010). For the correlation coefficient *r*, small effects were defined as 0.1, medium effects were 0.3, and large effects were 0.5 (Ellis 2010). The internal consistency of the subscales was examined using Cronbach’s α. An α value > 0.70 was considered satisfactory, and α values ≥ 0.90 were considered excellent (see Additional file 1 for Cronbach’s α values.).

Data were analysed using IBM SPSS statistics for Windows version 25 (Armonk, NY, USA: IBM Corp). Median regression was performed using STATA version 15 (StataCorp, College Station, Texas, USA).

## Results

### Sample characteristics

Table 1 presents the maternal and infant characteristics of the two samples.

**Table 1:** Characteristics of mothers and infants in the BD and the non-clinical sample

Variable	BD sample N = 26		Non-clinical sample N = 28		p value *significant
Maternal age at inclusion, in years, mean ± SD; range	30.5 ± 4.7; 22–37		33.5 ± 5.1; 27–44		0.03^*^
	n	%	n	%	
Parity					0.43
Primiparous	13	50	17	61	
Multiparous	13	50	11	39	
Completed education					< 0.001*
Primary school	8	31	1	4	
Secondary school	5	19	4	14	
Bachelor’s degree	11	42	7	25	
Master’s degree	2	8	16	57	
Employment status when not pregnant					0.003*
Working full-time	12	46	21	75	
Working part-time ± receiving benefits	4	15	2	7	
Receiving benefits only	8	31	0		
Unemployed	1	4	1	4	
School	1	4	4	14	
Infant gender					0.95
Girl	10	38	11	39	
Boy	16	62	17	61	
Infant birth weight, in g, mean ± SD; range	3632 ± 507; 2905-5085		3692 ± 424; 2911–4715		0.64
Infant gestational age, in months, mean ± SD; range	39.5 ± 1.2; 37.2-41.6		40 ± 1.2; 37–42		0.17
Clinical characteristics of BD sample					
Primary diagnosis					
BD I	7	27	Not applicable		
BD II	19	73			
Symptom load^a^ at 3 months					
Euthymia	8	31	Not applicable		
Mild depressive	6	23			
Moderate depressive	5	19			
Severe depressive	4	15			
Hypomania	2	8			
Mixed state^b^ (mild)	1	4			
Symptom load^a^ at 12 months					
Euthymia	8	31	Not applicable		
Mild depressive	4	15			
Moderate depressive	6	23			
Severe depressive	6	23			
Mania^c^	1	4			
Mixed state^d^ (severe)	1	4			

^a^ Euthymia = IDS score 0–13 + YMRS score 0–7; Mild depressive = IDS score 14–21; Moderate depressive = IDS score 22–30; Severe depressive = IDS score 31–38. Hypomania = YMRS score 8–20

^b^ Actual score: IDS = 14, YMRS = 11.5

^c^ One mother in recovery from a manic episode with hospitalisation. Not assessed with IDS or YMRS

^d^ Actual score: IDS = 34, YMRS = 16

The non-clinical sample of mothers was significantly older, had higher level of education and employment status than the BD sample. The infants’ gestational age and birth weight were within the normal range for both samples, and there were no significant group differences.

Within the BD sample, the symptom load increased over time, with more women having moderate to severe affective symptoms at 12 months than at 3 months, 54% vs. 34%, respectively. No significant associations were found between concurrent symptom load and interaction quality.

### Mother–infant interactions at 12 months

**Fig. 1:**
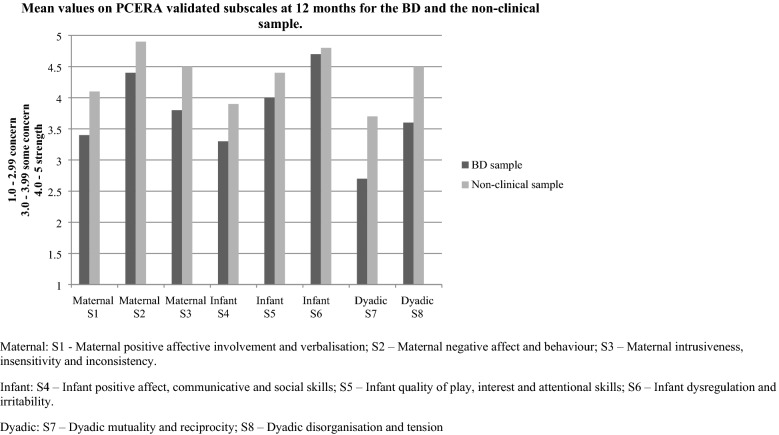
Mean values on PCERA validated subscales at 12 months for the BD and the non-clinical sample

Table 2 and Fig. 1 show the results for both samples on the validated 12-months PCERA scale. There were significant group differences with large effect sizes (Cohen’s *d* 0.97–1.78) on all subscales except on “Infant dysregulation and irritability” (S6), which had a small effect size (*r* 0.28).

**Table 2:** Interaction score comparisons (mean) between the BD and the non-clinical sample on PCERA subscales at 12 months

Subscale	BD sample n = 26	Non-clinical sample n = 28	Mean difference	Significance level	Cohen’s *d*
	Mean (sd) 95% CI	Mean (sd) 95% CI	95% CI		
S1					
Maternal positive affective involvement and verbalisation^a^	3.4 (0.57) 3.1–3.6	4.1 (0.51) 3.9–4.3	− 0.74 (− 1.03 to − 0.44)	< 0.001^*^	1.36
S2					
Maternal negative affect and behaviour^a^	4.4 (0.54) 4.2–4.6	4.9 (0.22) 4.8–5.0	− 0.49 (− 0.71 to − 0.27)	< 0.001^*^	1.19
S3					
Maternal intrusiveness, insensitivity and inconsistency^a^	3.8 (0.49) 3.6–4.0	4.5 (0.37) 4.4–4.6	− 0.68 (− 0.92 to − 0.45)	< 0.001^*^	1.57
S4					
Infant positive affect, communicative and social skills^a^	3.3 (0.68) 3.0–3.6	3.9 (0.61) 3.7–4.2	− 0.63 (− 0.98 to − 0.28)	0.001^*^	0.97
S5					
Infant quality of play, interest and attentional skills^a^	4.0 (0.40) 3.8–4.1	4.4 (0.37) 4.3–4.6	− 0.43 (− 0.64 to − 0.22)	< 0.001^*^	1.12
S6					
Infant dysregulation and irritability^b^	4.7 (4.5, 4.8) 4.6–4.8	4.9 (4.7, 5.0) 4.6–4.9	*	0.04^*^	0.28^c^
S7					
Dyadic mutuality and reciprocity^a^	2.7 (0.74) 2.4–3.0	3.7 (0.76) 3.4–4.0	− 1.03 (− 1.44 to − 0.63)	< 0.001^*^	1.38
S8					
Dyadic disorganisation and tension^a^	3.6 (0.57) 3.4–3.9	4.5 (0.41) 4.4–4.7	− 0.88 (− 1.14 to − 0.61)	< 0.001^*^	1.78

^a^Independent samples t-test

^b^Non-parametric test, Mann–Whitney U test, because of non-normal distribution of data. The results are presented as median values with interquartile range and range

^c^The effect size for Mann–Whitney U test: $r = \frac{Z}{\sqrt N }$^*^ Statistically significant results

#### Maternal subscales

The BD sample scored significantly lower than the non-clinical sample on all three maternal subscales (S1–S3). No confounding effects were found for “Maternal positive affective involvement and verbalisation” (S1). The associations remained significant after adjusting for maternal employment status on “Maternal negative affect and behaviour” (S2), (adjusted mean difference [∆_mean_]: − 0.37, 95% confidence interval [CI] − 0.61 to − 0.14, *p *= 0.002) and maternal age on “Maternal intrusiveness, insensitivity and inconsistency” (S3), (adjusted ∆_mean_: − 0.61, 95% CI − 0.84 to − 0.37, *p* < 0.001).

#### Infant subscales

The BD sample scored significantly lower than the non-clinical sample on all three infant subscales (S4–S6). The associations remained significant after adjusting for maternal employment status on all subscales (“Infant positive affect, communicative and social skills” [S4]: adjusted ∆_mean_: − 0.41, 95% CI − 0.78 to − 0.04, *p *= 0.029; “Infant quality of play, interest and attentional skills” [S5]: adjusted ∆_mean_: − 0.28, 95% CI − 0.50 to − 0.07, *p* = 0.01; “Infant dysregulation and irritability” [S6]: adjusted median difference: − 0.17, 95% CI − 0.32 to − 0.02, *p* = 0.02).

#### Dyadic subscales

The BD sample scored significantly lower than the non-clinical sample on both dyadic subscales (S7, S8). The associations remained significant after adjusting for maternal employment status (“Dyadic mutuality and reciprocity” [S7]: adjusted ∆_mean_: − 0.84, 95% CI − 1.28 to − 0.40, *p *< 0.001; “Dyadic disorganisation and tension” [S8]: adjusted ∆_mean_: − 0.72, 95% CI − 1.00 to − 0.44, *p* < 0.001).

### Development of mother–infant interactions from 3 to 12 months

**Fig. 2:**
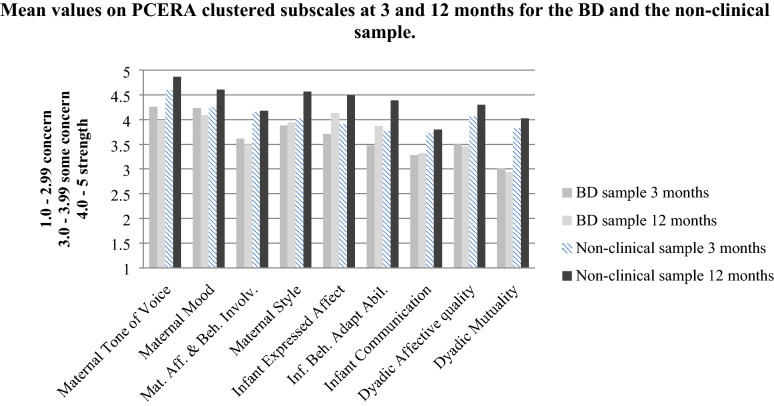
Mean values on PCERA clustered subscales at 3 and 12 months for the BD and the non-clinical sample

Table 3 demonstrates within- and between-group changes, based on the group mean values at 3 and 12 months on the nine clustered subscales. The group mean values at 3 and 12 months are also illustrated in Fig. 2.

**Table 3:** Mean values on PCERA-clustered subscales at 3 and 12 months and within- and between-group changes in the BD and the non-clinical sample from 3 to 12 months

Outcome variable	Sample	Mean (sd)	Mean (sd)	Mean within group change	Mean between group change
	size, n	at 3 months^a^	at 12 months^a^	3–12 months^b^ (95% CI); p-value	3–12 months^a^ (95% CI); p-value
Maternal tone of voice					
BD sample	26	4.26^#^ (0.41)	3.99^¤^ (0.49)	− 0.27 (− 0.45 to − 0.09); 0.006^*^	− 0.54 (− 0.84 to − 0.23); 0.01^*^
Non-clinical sample	28	4.60 (0.51)	4.86 (0.27)	0.27 (0.52 to 0.02); 0.04^*^	
Mother’s characteristic mood					
BD sample	26	4.23 (0.31)	4.09^¤^ (0.43)	− 0.14 (− 0.32 to 0.05); 0.14	− 0.49 (− 0.76 to − 0.22); 0.01^*^
Non-clinical sample	28	4.26 (0.50)	4.61 (0.23)	0.35 (0.55 to 0.15); 0.001^*^	
Maternal affective and behavioural involvement					
BD sample	26	3.61^#^ (0.46)	3.50^¤^ (0.56)	− 0.11 (− 0.34 to 0.12); 0.34	− 0.13 (− 0.54 to 0.28); 0.53
Non-clinical sample	28	4.15 (0.70)	4.18 (0.57)	0.02 (0.36 to − 0.32); 0.90	
Maternal style					
BD sample	26	3.88 (0.36)	3.94^¤^ (0.47)	0.05 (0.26 to − 0.15); 0.60	− 0.50 (− 0.84 to − 0.15); 0.005^*^
Non-clinical sample	28	4.02 (0.72)	4.57 (0.30)	0.55 (0.83 to 0.27); < 0.001^*^	
Infant expressed affect and characteristic mood					
BD sample	26	3.71 (0.58)	4.13^¤^ (0.43)	0.42 (0.67 to 0.18); 0.002^*^	− 0.16 (− 0.50 to 0.18); 0.36
Non-clinical sample	28	3.91 (0.64)	4.49 (0.44)	0.58 (0.83 to 0.33); < 0.001^*^	
Infant behavioural and adaptive abilities					
BD sample	26	3.48 (0.57)	3.87^¤^ (0.43)	0.40 (0.64 to 0.15); 0.003^*^	− 0.24 (− 0.64 to 0.15); 0.22
Non-clinical sample	28	3.76 (0.72)	4.39 (0.39)	0.64 (0.95 to 0.32); < 0.001^*^	
Infant communication					
BD sample	26	3.28 (0.77)	3.32^¤^ (0.69)	0.04 (0.43 to − 0.35); 0.84	− 0.03 (− 0.57 to 0.50); 0.90
Non-clinical sample	28	3.73 (0.86)	3.80 (0.53)	0.07 (0.45 to − 0.31); 0.70	
Dyadic affective quality					
BD sample	26	3.5^#^ (0.65)	3.46^¤^ (0.68)	− 0.04 (0.26 to − 0.34); 0.79	− 0.28 (− 0.67 to 0.11); 0.16
Non-clinical sample	28	4.06 (0.64)	4.30 (0.47)	0.24 (0.51 to − 0.03); 0.08	
Dyadic mutuality					
BD sample	26	3.01^#^ (0.63)	2.94^¤^ (0.63)	− 0.07 (0.23 to − 0.36); 0.64	− 0.26 (− 0.78 to 0.25); 0.30
Non− clinical sample	28	3.82 (0.88)	4.02 (0.65)	0.20 (0.63 to − 0.23); 0.36	

^a^Independent samples t-test

^b^Paired samples t-test

^#^Statistically significant mean group difference at 3 months, *p *< 0.05. ^¤^ Statistically significant mean group difference at 12 months, *p *< 0.05. ^*^Statistically significant results

#### Maternal subscales from 3 to 12 months

On the subscale “Maternal tone of voice”, the BD sample had a significant negative change, whereas the non-clinical sample had a significant positive change. Thus, the groups developed in opposite directions, resulting in a significant mean between group change.

On the subscales “Mother’s characteristic mood” and “Maternal style”, the BD sample had no changes, whereas the non-clinical sample had significant positive changes, resulting in significant mean between group changes.

On the subscale “Maternal affective and behavioural involvement”, there were neither significant within nor between group changes from 3 to 12 months.

There were no confounding effects of any of the possible confounders on the maternal subscales.

#### Infant subscales from 3 to 12 months

On the subscales “Infant expressed affect and characteristic mood” and “Infant behavioural and adaptive abilities”, both samples had significant positive changes, and there were no significant mean between group changes.

On the subscale “Infant communication”, there were neither significant within nor between group changes from 3 to 12 months.

A confounding effect of maternal employment status was found on “Infant communication”, but it did not change the results. No other confounding variables were revealed for the infant subscales.

#### Dyadic subscales from 3 to 12 months

On the subscales “Dyadic affective quality” and “Dyadic mutuality”, there were neither significant within nor between group changes from 3 to 12 months.

A confounding effect of maternal employment status was found on “Dyadic affective quality”, but it did not change the results. No other confounding variables were found for the dyadic subscales.

## Discussion

The current study contributes to knowledge on early environmental influences for bipolar offspring, as expressed in mother–infant interactions in the first year of life. We investigated the patterns and development of mother–infant interactions from 3 to 12 months in dyads in which the mothers had BD, compared to dyads in which the mothers had no mental disorders.

In line with our anticipation, there were significant group differences at 12 months in all three domains that were studied: maternal behaviour, infant behaviour and dyadic coordination, with more interaction difficulties in the BD group. The majority of concerning interaction behaviours at 3 months was also found at 12 months in the BD group. Below, we discuss the main findings within the different interaction domains. Within each domain, we first present the main findings on the mother–infant interaction patterns at infant age 12 months. Then we discuss the development within and between the groups from infant ages 3 to 12 months.

### Dyadic coordination

The results showed strongest support for BD dyads having concerning interaction behaviours in the domain of dyadic coordination.

At 12 months, the largest group mean differences across all subscales were found in the dyadic subscales “Dyadic mutuality and reciprocity” (S7) and “Dyadic disorganisation and tension” (S8), implying that the dyadic domain differentiated the BD sample from the non-clinical sample the most. Furthermore, on “Dyadic mutuality and reciprocity” (S7) the BD group mean was in the area of concern, meaning that these interaction behaviours were evidently problematic for the BD dyads. On “Dyadic disorganisation and tension” the BD group mean was in the area of some concern (S8), thus demonstrating challenges. The respective group means for the non-clinical sample were in the area of some concern (S7) and strength (S8) (Table 2).

Although none of the samples demonstrated any significant change on the subscales “Dyadic affective quality” and “Dyadic mutuality” from 3 to 12 months, the BD dyads had sustained, and significantly lower group means than the comparison dyads (Table 3). A closer inspection of the dyadic scales at 3 and 12 months, revealed that the BD sample had mean values in the area of concern on the variables “flat, empty, constricted dyadic affect”, “mutual enthusiasm, joyfulness” and “reciprocity”, whereas the mean values on dyadic “frustration, anger, hostility” and “tension, anxiety” were in the area of strength (see Tables 2.1 and 2.2 in Additional file 2). Hence, in the first year, the main dyadic challenge for the mothers and infants was to “find” each other and share a positive “rhythmic dance”, rather than their exchanges being affected by anger and tension.

Importantly, the establishment of dyadic coordination and synchrony seems to be of particular significance during a sensitive period between two and nine months (Feldman, 2015), which aligns with the timespan of the current study. There is a biobehavioural shift in infant development at 2–3 months, when infants become “ready” to participate in recurring patterns of coordinated social “give-and-takes” (Zeanah et al. 1997). As these provide critical building blocks for infants’ evolving social capacities and emotion regulation, poor dyadic coordination may have negative developmental influence for the child (Feldman 2007a, b, 2015; Granat et al. 2017; Leclere et al. 2014; Weinberg and Tronick 1997). Furthermore, the early impairments in dyadic coordination may be associated with later difficulties with cooperation and resolution of conflict observed among BD dyads (Kochanska et al. 1987). This should be explored in prospective investigations of the mother–infant interactions beyond the first 12 months.

The significant group differences in the dyadic domain in the current study support previously reported statistical trends of dyadic difficulties among BD dyads at 12 months (Logsdon et al. 2015). Differences in statistical strengths may in part be related to different measurements. Whereas the PCERA is evaluated to have good sensitivity and discriminant validity (Clark 1985, 2006, 2009, 2010; Clark 1999; Lotzin et al. 2015), even with small sample sizes (Minde et al. 1994; Savonlahti et al. 2005), Logsdon et al. (2015) discuss whether their measurements were not sensitive enough to detect subtle differences in mother–infant interactions.

### Maternal interaction behaviour

The BD sample scored significantly lower than the non-clinical sample on all maternal subscales at 12 months (S1–S3) (Table 2). When applying the PCERA areas of concern/strength in the interpretation of the findings, the group differences appear most consequential on the subscale “Maternal positive affective involvement and verbalisation” (S1). On this subscale, the BD mothers had their lowest group mean value, in the area of some concern (vs. area of strength for the non-clinical sample), revealing challenges with expression of positive affect, infant attuned verbalisations and involvement. Contrasting, on the subscale “Maternal negative affect and behaviour” (S2), the BD sample had a mean value well within the area of strength, showing that the BD mothers expressed little negative affect, such as anger, disapproval and irritability (Table 2).

From 3 to 12 months, the BD sample showed no significant changes on the subscales “Maternal affective and behavioural involvement”, “Mother’s characteristic mood” and “Maternal style”, whereas the non-clinical sample had significant positive changes on the two latter subscales (Table 3). We find the BD sample’s lack of change on “Maternal affective and behavioural involvement” concerning. Here, the BD sample’s mean value was significantly lower than the non-clinical sample’s mean value at both 3 and 12 months (area of some concern vs. area of strength), and it was the lowest mean value across the maternal subscales at both time points (Table 3). Hence, these findings indicate continuous challenges among the BD mothers in expression of affective and behavioural involvement, such as social initiatives, reading infant cues and responding contingently, mirroring, structuring and mediating.

Notably, the affective quality of “Maternal tone of voice” developed in opposite directions for the two groups (Table 3). Even though the non-clinical sample had a significantly warmer and more emotional tone of voice than the BD sample at 3 months (area of strength), it became even more so at 12 months. In contrast, the tone of voice in the BD sample changed significantly towards more flatness and less emotionality. Although the BD group mean changed to just beneath the area of strength, we find the decline worthy of some reflections. First, the quality of voice and its’ emotional prosody is regarded as an indicator of the individual’s underlying affective state (Belin et al. 2004; Scherer 1986, 1995, 2003). For instance, studies have shown that the tone of voice and speech patterns change with depressive mood (Cannizzaro et al. 2004; Ellgring and Scherer 1996; Garcia-Toro et al. 2000). The severity of depressive symptomatology did increase in our BD sample in the first year. At 3 months, 34% of the BD mothers had moderate to severe depressive symptoms, whereas this increased to 46% at 12 months (Table 1). It is possible, or even likely, that the significant change in tone of voice is a reflection of the deterioration in maternal affective state.

Second, qualities and characteristics of maternal voice and speech are important in mother–infant interactions (Saint-Georges et al. 2013). A flat tone of voice with reduced emotionality lacks the acoustic cues that are ingredients in infant directed speech—i.e. motherese or “baby-talk”—an emotional form of speech attuned to the infant with specific linguistic, prosodic and affective characteristics (Saint-Georges et al. 2013). Still, tone of voice is only one aspect of motherese. Other aspects of motherese are captured by the maternal variable “quality of verbalisation”, which had a group mean value in the border area of concern/some concern at 12 months (group mean value 3.1). Together, these data suggest challenges in motherese in the BD sample. Since motherese has been found to promote different infant behaviours, such as attention (Senju and Csibra 2008; Werker and McLeod 1989; Zangl and Mills 2007), responsiveness (Saint-Georges et al. 2013; Werker McLeod 1989), language learning (Golinkoff et al. 2015; Thiessen et al. 2005) and infant expression of positive affect (Saint-Georges et al. 2013), the current results on maternal tone of voice and quality of verbalisations may signal enhanced developmental risk for the infants. Reduced motherese has also been found among mothers with unipolar depression (Bettes 1988; Herrera et al. 2004; Kaplan et al. 2001, 2002).

Overall, our results concur with reported trends (Logsdon et al. 2015) and significant findings (Hipwell et al. 2000) of less sensitive interaction behaviours at 12 months among BD mothers than among control mothers.

A number of factors may explain the maternal interaction difficulties in the BD group. For instance, adverse social circumstances may co-exist with severe mental illness and have negative impact on maternal interaction behaviours (Abel et al. 2005; Lewin and Templin 2016). However, the women in our BD sample were satisfied with their life situation (i.e., housing and economy), most felt support from their cohabitating partner, and several also had access to assistance from their family network (Anke et al. 2019b). The education level was on par with the general Norwegian population, and 46% of the BD women worked full-time. Together, these characteristics suggest resourcefulness, and are at odds with the adverse social circumstances hypothesis.

Affective episode is another factor that may influence maternal interaction behaviours. We found no impact of concurrent symptom load on interaction measures. This corresponds with findings in other studies on parent-infant interaction and mood disorders (Anke et al. 2019a; Campbell et al. 1995; Forbes et al. 2004). However, it is important to emphasise that concurrent symptom load is a momentary measure. It informs us of the potential influence of mood during interaction sessions, but does not capture the full impact of the illness during the first postpartum year. Large variations in illness course and lack of statistical power did not allow us to explore this further. Thus, we cannot rule out that affective episodes may have an effect on maternal interaction behaviours. Also, it is conceivable that more extreme concurrent mood deviations than those observed in our study, may have an immediate impact on interactions.

In addition, mood disorders are associated with atypical neural processing of emotion in brain areas that overlap with maternal sensitivity networks (Bjertrup et al. 2019). For example, mothers with unipolar depression display more dampened neural responses to infant signals than controls (Bjertrup et al. 2019). It is an intriguing question whether corresponding deviations may apply to mothers with BD during interaction with their infants, given that individuals with BD have shown impairments in emotion perception and processing across different phases of the illness (Samame 2013; Samame et al. 2012; Vaskinn et al. 2017). However, the question has not yet been investigated and motivates further studies.

### Infant interaction behaviour

The BD sample scored significantly lower than the non-clinical sample on all infant subscales at 12 months (S4–S6) (Table 2). Following the same logic as above, when applying the PCERA areas of concern/strength in the interpretation of the findings, the group differences appear most consequential on the subscale “Infant positive affect, communicative and social skills” (S4). On this subscale, the BD infants had their lowest mean value, in an area of some concern, whereas the mean values for the other two subscales (S5–S6) were in an area of strength. In particular, on the subscale “Infant dysregulation and irritability” (S6), the BD sample had a high mean value in an area of strength (Table 2). Thus, similar to their mothers, the BD infants displayed little negative affect and irritability.

From 3 to 12 months, both infant samples showed significant positive change on subscales “Infant expressed affect and characteristic mood” and “Infant behavioural and adaptive abilities” (Table 3). Given that mother–infant interactions influence the infant’s development (Crockenberg and Leerkes 2000; Nelson and Bosquet 2000; Tronick 2007), and that infants are highly sensitive to maternal affective state (Cohn and Tronick, 1983, 1989), the BD infants’ positive change seems counterintuitive in relation to the maternal behaviours of concern (i.e. subdued positive affect and underinvolvement). However, a closer inspection of the scales in question, revealed that the group mean values for the variables of “expressed positive affect”, “happy, cheerful mood”, “social initiatives” and “social responses”, were all in the range of 2.6–3.3 (i.e. area of concern to area of some concern) at both 3 and 12 months (see Tables 3.1 and 3.2 in Additional file 3). Hence, the BD infants displayed corresponding challenges with expression of positive affect and interactional involvement as the BD mothers. Basically, it is difficult for infants to build positive arousal and maintain positive affect without adult assistance (Feldman, 2003, 2007b; Weinberg and Tronick 1997). The current findings give support to previously reported trends of decreased infant expressivity (Hipwell et al. 2000) and findings of avoidant infant behaviour (Gaensbauer et al. 1984).

The BD infants’ positive change was best demonstrated on the variables of “alertness, interest” and “robustness”, and a decrease in “expressed negative affect”. On these variables, the BD infants reached high group mean values in an area of strength from 3 to 12 months. The data also provided few signs of “emotional lability”, “anxious” or “irritable mood” among the BD infants, as these variables showed high group means in an area of strength from 3 to 12 months (see Tables 3.1 and 3.2 Additional file 3).

Taken together, several infants in the BD sample showed subdued affect and little social turn-taking, but were toy oriented with interest and alertness. To what extent the BD infants’ state and toy involvement reflect a genuine positive development or a defensive self-regulatory behaviour, because of maternal underinvolvement, is unclear (Granat et al. 2017; Hart et al. 1999; Hipwell et al. 2000; Weinberg and Tronick, 1997).

None of the infant samples demonstrated change on the subscale “Infant communication”, from 3 to 12 months. Notable, the BD infants’ mean value of 3.3 was the lowest group mean value across the infant subscales and significantly lower than for the comparison infants at 12 months. This comprised both the clustered scale “Infant communication” (Table 3) and the validated scale “Infant positive affect, communicative and social skills” (S4) (Table 2). The data thus imply weak social communication among the BD infants, which also has been reported among infants of depressed mothers (Field 1995; Granat et al. 2017; Tronick and Reck 2009). Communication is a collaborative process where weak infant communication may result from insufficient maternal responsiveness and motherese in interactions (Bornstein et al. 2008; Golinkoff et al. 2015; Tamis-LeMonda et al. 2014).

We recognise that in the above discussion of the BD infants’ interaction behaviours, we tilt towards interpreting the infants’ difficulties as resulting from insufficient maternal support, e.g. maternal underinvolvement in the interactions. However, we cannot rule out the possibility that constitutional factors contribute to the infants’ difficulties, which in turn may have an adverse effect on the mothers’ behaviours. The possible influence of constitutional factors is an unanswered and complex question pertaining to infants of mothers with inheritable mental disorders, such as BD and schizophrenia (Johnson et al. 2014; Harder et al. 2015; Wan et al. 2007, 2008). On the other hand, regardless of the “origin” of the observed infant difficulties, the infant is highly dependent on attuned developmental support in mother–infant interactions.

### Interaction patterns across the three domains

To summarise, the BD sample displayed interaction patterns of subdued positive affect and mutual underinvolvement across the three interaction domains. This condition implies a risk for the dyads being “trapped” in vicious circles. For instance, maternal affective and behavioural underinvolvement make it difficult for the infant to achieve social connectedness, and dyadic reciprocity is undermined (Granat et al. 2017; Weinberg & Tronick, 1997).

The current interaction patterns in the BD sample resemble patterns of dyads with depressed mothers who have downcast affect and a withdrawn behavioural style (Field 1984; Field et al. 2003; Hart et al. 1999; Malphurs et al. 1996; Tronick and Reck 2009). As a considerable body of evidence demonstrate the adverse short- and long-term consequences of maternal depression on child development (Goodman et al. 2011; Stein et al. 2014) the resemblance is noteworthy, given that non-optimal interaction patterns are assigned an important explanatory role (Goodman and Gotlib 1999). It is further suggested that depressive maternal interaction behaviours of either a withdrawn or intrusive style, yield different types of developmental risk for the child (Field et al. 2003; Hart et al. 1999). It is proposed that a withdrawn style increases the risk for impairments in affective and social-emotional development since it entails little affect sharing and maternal regulation (DeMulder and Radke-Yarrow 1991; Granat et al. 2017; Hart et al. 1999).

## Clinical implications

Given that BD is a severe mental illness with a high risk of postpartum illness relapse, we underscore the importance of addressing both the mother’s needs and the mother–infant interactions. This is in agreement with treatment approaches for mothers with postpartum depression and their infants (MacBeth et al. 2015; Nylen et al. 2006; Puckering et al. 2010).

Thus, early detection and treatment of BD postpartum mood deviations is pivotal, including providing support for the mother in her experience of distress (Puckering et al. 2010). Such interventions contribute to valuable premises for positive mother–infant interactions. However, studies on maternal depression indicate that alleviation of maternal symptoms alone is not sufficient for positive outcome for the infant and mother–infant interactions (Logsdon et al. 2009; Nylen et al. 2006; Puckering et al. 2010). The mother–infant interactions need to be explicitly targeted to promote resilient infant development. Furthermore, well-functioning interactions may reinforce the mother’s sense of competence (Stern 1995; Weatherston and Fitzgerald 2010), which is a particularly important matter for vulnerable mothers.

Therefore, we suggest interaction interventions that sensitise mothers to their infant’s cues on a micro-level. Either through in vivo guidance or through viewing video-recorded interactions, the mothers can get detailed feedback about their infant’s communicative cues and be guided in attuned contingent responses. Such approaches may strengthen dyadic coordination and reciprocity. Based on the current findings, the feedback should be attentive to positive affect sharing and mutual involvement. Notably, video feedback guidance has proven effective in enhancing parent-infant interactions in the context of maternal depression (Høivik et al. 2015; Van Doesum et al. 2008) and demonstrated positive effect on maternal depressive symptoms (Høivik et al. 2015).

## Strengths and limitations

A main strength of the current study was the inclusion of both maternal and infant behaviour and their dyadic coordination for interaction assessments. Additionally, these behaviours were investigated at two time points, which allowed for a more comprehensive understanding of interaction patterns during the infants’ first year of life.

The study is subject to several limitations. First, the coders were not naïve to the mothers’ BD status. Counteracting possible biases, the variables in PCERA are strictly operationalised in the manual, with extensive descriptions to enhance the precision of ratings (e.g., frequency, duration and intensity). Furthermore, the coders are highly experienced, trained and certified for reliability and have no affiliation with our research milieu on bipolar disorder—perinatal mental health—infant mental health. Second, almost all BD dyad interactions were carried out in the participants’ homes at both time points. In contrast, all healthy dyad interactions were recorded in a professional setting. Given the more vulnerable situation of the BD sample, we found it proper to let mothers with BD choose the location. However, it is likely that the more optimal location for the BD dyads may have reduced the intergroup differences somewhat.

Third, close to all mothers confirmed the representativeness of the interaction sessions. However, there were a few exceptions in the BD group (one at 3 months, and two at 12 months). We assess that this may have had some, but limited impact on the main findings.

Fourth, we explored the possibility of assessing the effect of psychopharmacological treatment, and other interventions from mental health services, on mother–infant interactions in the BD group. Because of too small groups, and too large internal variations in different treatments, this was not feasible.

Fifth, the women in the non-clinical sample were older, had a higher level of education and employment status than the women in the BD sample. A confounding effect was found for maternal employment status on some of the subscales, and for maternal age on one subscale, and these were adjusted for. The adjustments did not change the results. Furthermore, the BD sample resembles the general population in Norway. Thus, both samples may be skewed towards the resourceful end of the populations they represent. For the sake of comparison, the resourcefulness of the women with BD was beneficial, but it may limit the generalisation of the findings to less resourceful women with BD.

Sixth, the relatively small sample size implies that the findings need to be interpreted with some caution. A small sample size increases the width of confidence intervals and limits generalisability.

Finally, a large number of statistical tests increase the likelihood of one or more false positives findings. Nevertheless, we have chosen not to adjust for multiple comparisons as correcting for type I errors cannot be done without inflating type II errors (Perneger 1998).

The limitations of the current study, and the general scarcity of studies on the subject matter, underscore the need for more studies. This includes studies on less resourceful dyads, and dyads with single mothers. Studies on the developmental trajectories of mother–infant interactions beyond the first year are important, as existing literature indicates increasing difficulties with child age, including more conflicted interactions (DeMulder and Radke-Yarrow 1991; Gaensbauer et al. 1984; Kochanska et al. 1987). We also suggest studies of father-infant interaction when the mother has BD to investigate whether the infant’s non-optimal interaction behaviour may be relationship-specific. Also, it is conceivable that well-functioning father-infant interactions may moderate risk in mother–infant interactions.

## Conclusion

We found more interactional challenges in the first year among dyads in which the mothers had BD, compared to dyads in which the mothers had no mental disorder. Subdued expression of positive affect and mutual underinvolvement represented core challenges in maternal and infant behaviours in the BD dyads. Continuous difficulties with dyadic coordination and reciprocity were the most concerning interaction behaviours at 3 and 12 months.

On the positive side, there was little expression of negative affect or tension in maternal, infant and dyadic behaviour, and some positive changes in infant behaviour from 3 to 12 months.

Altogether, we conclude that less optimal quality of mother–infant interactions in the context of maternal BD may heighten the risk of an unfavourable developmental pathway for the bipolar offspring.

## Supplementary information

**Additional file 1.** Description of PCERA subscales used for analyses.

**Additional file 2.** Tables on mean values of dyadic variables for the BD sample at 3 and 12 months.

**Additional file 3.** Tables on mean values of infant variables for the BD sample at 3 and 12 months.

## Data Availability

The datasets generated and analysed for the current study (film recordings) will not be shared or made publicly available since participants may be identifiable. Request of permission to access data may be sent to the corresponding author.

## Abbreviations

BD: Bipolar disorder
PCERA: Parent–Child Early Relational Assessment
IDS: Inventory of Depressive Symptomatology
YMRS: Young Mania Rating Scale
