# Supplementary material for: Bipolar offspring and mothers: interactional challenges at infant age 3 and 12 months—a developmental pathway to enhanced risk?
Source: Int J Bipolar Disord. 2020 Aug 31;8:27. doi: 10.1186/s40345-020-00192-3 (PMC7459000; doi:10.1186/s40345-020-00192-3)
Supplement: Supplementary file 1 — Additional file 1. Description of PCERA subscales used for analyses. [file 40345_2020_192_MOESM1_ESM.docx]

**Additional file 1**. Further description of the organisation of PCERA variables into subscales (see Methods section: PCERA subscales used for analysis).

Two variables from the original validated PCERA subscales for 12 months were excluded in the current study. Variable 20, maternal “Contingent responsivity to child’s perceived negative and/or unresponsive behaviour” was not possible to rate as no such behaviour occurred during the interactions. Variable 48, infant “Persistence” was excluded on recommendation from the coders since it is difficult to evaluate persistence in a situation that allows free, spontaneous play. Thus, the final subscales contained 21 maternal, 19 infant and 8 dyadic variables (see Table 1.1 below).

In the organisation of PCERA clustered subscales for 3-12 months, the maternal subscales “Maternal affect”, “Mother’s expressed attitude toward child” and the infant scale “Activity level” were excluded because of low internal consistency at 3 and/or 12 months (Cronbach’s α < 0.60). Furthermore, the following three single variables were excluded. Variable 20, maternal “Contingent responsivity to child’s perceived negative and/or unresponsive behaviour” was not possible to rate as no such behaviour occurred. Variable 13, maternal “Quality and amount of physical contact: Positive” was infrequent/non-existent in both groups at 12 months. This finding is likely attributable to the play situation. Therefore, low scores (1 or 2) could be non-valid with regard to the “true” occurrence of positive physical contact in the mother-infant interactions. Finally, variable 51, infant “Consolability, soothability” was excluded, as it was not possible to rate in the absence of necessary soothing during the interaction. The final clustered subscales comprised four maternal, three infant and two dyadic scales, which include 23 maternal, 18 infant and 8 dyadic variables (see Table 1.2 below).

**Supplementary Table 1.1.** Validated PCERA subscales for interactions at infant age 12 months.

| Subscale | Variables included in subscales | Cronbach’s alpha  BD sample Non-clinical sample |
| --- | --- | --- |
| S1-  Maternal^1^ positive affective involvement and verbalisation | 2)^2^  Flat, unemotional, constricted tone of  voice  4) Expressed positive affect  7) Depressed mood  12) Enjoyment, pleasure  15) Amount and quality of visual contact  with child  16) Amount of verbalisation  17) Quality of verbalisation  18) Social initiative  21) Structures and mediates environment  24) Mirroring  26) Resourcefulness, creativity | 0.94 0.94 |
| S2-  Maternal negative affect and behaviour | 1) Annoyed, angry tone of voice  5) Expressed negative affect  6) Irritable/ frustrated/angry mood  11) Displeasure, disapproval, criticism  20)^3^ Contingent responsivity to child’s perceived negative and/or unresponsive behaviour. | 0.88 0.79 |
| S3-  Maternal intrusiveness, insensitivity and inconsistency | 8) Anxious mood  14) Quality and amount of physical  contact: Negative  16) Amount of verbalisation  21) Structures and mediates environment  22) Sensitivity and responsivity  25) Flexibility/Rigidity  27) Intrusiveness  28) Consistency, predictability | 0.85 0.85 |
| S4-  Infant positive affect, communicative and  social skills | 30) Expressed positive affect  32) Happy, pleasant, content, cheerful  mood  33) Apathetic, withdrawn, depressed  mood  36) Sober/serious mood  39) Social behaviour of infant - initiates  55) Visual contact  56) Communicative competence  57) Readability | 0.93 0.93 |
| S5-  Infant quality of play, interest and attentional skills | 33) Apathetic, withdrawn, depressed  mood  38) Alertness, interest  44) Motoric competence and quality  45) Quality of exploratory play  46) Attentional abilities  47) Robustness  48)^3^ Persistence  50) Self-regulation, organisational  capacities  56) Communicative competence  57) Readability | 0.85 0.85 |
| S6-  Infant dysregulation  and irritability | 31) Expressed negative affect  35) Irritable/frustrated/ angry mood  37) Emotional lability  43) Assertion/aggressivity  49) Impulsivity  50) Self-regulation, organisational  capacities | 0.57 0.90 |
| S7-  Dyadic mutuality and reciprocity | 59) Flat, empty, constricted  61) Mutual enthusiasm, joyfulness,  enjoyment, a sense of dyadic “Joie de  Vivre”  63) Reciprocity  65) Goodness of fit | 0.92 0.91 |
| S8-  Dyadic tension | 58) Frustrated, angry, hostile  60) Tension, anxiety  62) Joint attention, activity  64) Organisation, regulation of  interactions  65) Goodness of fit | 0.88 0.75 |

^1^ In the manual the word “parental” is used instead of “maternal” (Clark, 2010).

^2^ Variable number in manual (Clark, 2010).

^3^ Variable excluded from statistical analysis.

**Supplementary Table 2.** PCERA clustered subscales (with variables applicable for 3 and 12 months).

| Subscale | Variables included in subscales | Cronbach’s alpha  3 months 12 months |
| --- | --- | --- |
| Maternal^1^ scales |  |  |
| Tone of voice | 1)^2^ Annoyed, angry tone of voice  2) Flat, unemotional, constricted tone  of voice  3) Warm, kind tone of voice | 0.63 0.78 |
| Maternal affect^3^ | 4) Expressed positive affect  5) Expressed negative affect | 0.48 0.49 |
| Mother’s characteristic mood | 6) Irritable/frustrated/angry mood  7) Depressed mood  8) Anxious mood  9) Cheerful, animated, enthusiastic  mood, “Joie de Vivre”  10) Hypomanic mood/behaviour | 0.63 0.75 |
| Mother’s expressed attitude toward child^3^ | 11) Displeasure, disapproval, criticism  12) Enjoyment, pleasure | 0.43 0.76 |
| Maternal affective and behavioural involvement | 13)^4^ Quality and amount of physical  contact: Positive  14) Quality and amount of physical  contact: Negative  15) Amount and quality of visual contact  with child  16) Amount of verbalisation  17) Quality of verbalisation  18) Social initiative  19) Contingent Responsivity to child’s  positive and/or age-appropriate  behaviour  20)^4^ Contingent responsivity to child’s  perceived negative and/or  unresponsive behaviour  21) Structures and mediates environment  22) Mother reads child’s cues and  responds sensitively and  appropriately  23) Connectedness  24) Mirroring | 0.93 0.94 |
| Maternal style | 25) Flexibility/rigidity  26) Resourcefulness, creativity  27) Intrusiveness  28) Consistency, predictability  29) Evidence of behavioural  disturbances | 0.83 0.87 |
| Infant scales |  |  |
| Infant’s expressed affect and characteristic mood | 30) Expressed positive affect  31) Expressed negative affect  32) Happy, pleasant, content, cheerful  mood  33) Apathetic, withdrawn, depressed  mood  34) Anxious, tense, fearful mood  35) Irritable/frustrated/angry mood  36) Sober/serious mood  37) Emotional lability | 0.87 0.82 |
| Behaviour/adaptive abilities | 38) Alertness, interest  39) Social behaviour of infant - Initiates  40) Social behaviour of infant -  Responds  41) Avoiding, averting, resistance  46) Attentional abilities  47) Robustness  50) Self-regulation, organisational  capacities  51)^4^ Consolability, soothability | 0.91 0.81 |
| Activity level^3^ | 53) Passivity, lethargy  54) Hyperactivity/overactivity | <0 <0 |
| Communication | 55) Visual contact  56) Communicative competence  57) Readability | 0.84 0.82 |
| Dyadic scales |  |  |
| Affective qualitative of interaction | 58) Frustrated, angry, hostile  59) Flat, empty, constricted  60) Tension, anxiety  61) Mutual enthusiasm, joyfulness,  enjoyment, a sense of dyadic  “Joie de Vivre” | 0.82 0.84 |
| Mutuality | 62) Joint attention, activity  63) Reciprocity  64) Organisation, regulation of  interactions  65) Goodness of fit | 0.93 0.94 |

^1^ In the manual the word “parental” is used instead of “maternal” (Clark, 2010).

^2^ Variable number in manual (Clark, 2010).

^3^ Subscale excluded from statistical analysis, because of Cronbach’s α < 0.60.

^4^ Variable excluded from statistical analysis.
