# Supplementary material for: Bipolar offspring and mothers: interactional challenges at infant age 3 and 12 months—a developmental pathway to enhanced risk?
Source: Int J Bipolar Disord. 2020 Aug 31;8:27. doi: 10.1186/s40345-020-00192-3 (PMC7459000; doi:10.1186/s40345-020-00192-3)
Supplement: Supplementary file 2 — Additional file 2. Tables on mean values of dyadic variables for the BD sample at 3 and 12 months. [file 40345_2020_192_MOESM2_ESM.docx]

**Additional file 2.** Tables on mean values of dyadic variables for the BD sample at 3 and 12 months.

**Supplementary Table 2.1**. Mean values on PCERA clustered subscale “Dyadic affective quality” for the BD sample (n=26) at 3 and 12 months.

| Dyadic Variable | Mean value at 3 months | Mean value at 12 months |
| --- | --- | --- |
| Frustrated, angry, hostile | 4.54 | 4.69 |
| Flat, empty, constricted | 2.85 | 2.65 |
| Tension, anxiety | 4.15 | 4.19 |
| Mutual enthusiasm, joyfulness, enjoyment, a sense of dyadic “Joie de Vivre” | 2.46 | 2.31 |

**Supplementary Table 2.2**. Mean values on PCERA clustered subscale “Dyadic mutuality” for the BD sample (n=26) at 3 and 12 months.

| Dyadic Variable | Mean value at 3 months | Mean value at 12 months |
| --- | --- | --- |
| Joint attention, activity | 3.04 | 3.08 |
| Reciprocity | 2.54 | 2.46 |
| Organisation, regulation of interactions | 3.27 | 3.04 |
| Goodness of fit | 3.19 | 3.19 |
