# Supplementary material for: Bipolar offspring and mothers: interactional challenges at infant age 3 and 12 months—a developmental pathway to enhanced risk?
Source: Int J Bipolar Disord. 2020 Aug 31;8:27. doi: 10.1186/s40345-020-00192-3 (PMC7459000; doi:10.1186/s40345-020-00192-3)
Supplement: Supplementary file 3 — Additional file 3. Tables on mean values of infant variables for the BD sample at 3 and 12 months. [file 40345_2020_192_MOESM3_ESM.docx]

**Additional file 3.** Tables on mean values of infant variables for the BD sample at 3 and 12 months.

**Supplementary Table 3.1**. Mean values on PCERA clustered subscale “Infant’s expressed affect and characteristic mood” for the BD sample (n=26) at 3 and 12 months.

| Infant Variable | Mean value at 3 months | Mean value at 12 months |
| --- | --- | --- |
| Expressed positive affect | 2.58 | 3.04 |
| Expressed negative affect | 3.69 | 4.65 |
| Happy, pleasant, content, cheerful  mood | 2.73 | 3.31 |
| Apathetic, withdrawn, depressed mood | 3.65 | 3.88 |
| Anxious, tense, fearful mood | 4.42 | 4.81 |
| Irritable/frustrated/angry mood | 4.42 | 4.81 |
| Sober, serious mood | 3.42 | 3.46 |
| Emotional lability | 4.77 | 4.96 |

**Supplementary Table 3.2.** Mean values on PCERA clustered subscale “Infant behavioural and adaptive abilities” for the BD sample (n=26) at 3 and 12 months.

| Infant Variable | Mean value at 3 months | Mean value at 12 months |
| --- | --- | --- |
| Alertness/Interest | 3.73 | 4.81 |
| Social behaviour of infant - Initiates | 3 | 2.73 |
| Social behaviour of infant -  Responds | 2.85 | 3.12 |
| Avoiding, averting, resistance | 3.65 | 3.62 |
| Attentional abilities | 3.58 | 3.92 |
| Robustness | 3.77 | 4.81 |
| Self-regulation, organisational  capacities | 3.77 | 4.12 |
